# Behaviour of *Listeria monocytogenes* and Natural Microflora during the Manufacture of Riojano Chorizo (Spanish Dry Cured Sausage)

**DOI:** 10.3390/microorganisms9091963

**Published:** 2021-09-15

**Authors:** Elena Gonzalez-Fandos, Maria Vazquez de Castro, Alba Martinez-Laorden

**Affiliations:** Food Technology Department, CIVA Research Center, University of La Rioja, Madre de Dios 53, 26006 Logroño, Spain; mavadeca@gmail.com (M.V.d.C.); alba.mar.lao@outlook.es (A.M.-L.)

**Keywords:** food safety, dry sausages, foodborne pathogens, microbial survival, *Listeria monocytogenes*, RTE, ready-to-eat, meat products

## Abstract

Riojano chorizo is a dry cured sausage manufactured with traditional technologies without adding starter cultures at low temperatures. Its characteristics differ from other types of chorizo since sugars and nitrites are no added and processing temperatures are low- This work evaluates the behaviour of *Listeria monocytogenes* during the processing of inoculated Riojano chorizo as well as the natural microflora that can play a technological role or be of interest as indicators. The sausage mixture was inoculated with a cocktail of three selected strains of *L. monocytogenes* (CECT 932, CECT 934 and CECT 4032) (4 log_10_ CFU/g) and after processed following the traditional production method. Samples were taken before inoculation, after inoculation, after stuffing (day 0) and on days 6, 13, 21 and 28 of processing. *L. monocytogenes*, mesophiles, *Micrococcaceae*, lactic acid bacteria, *Enterobacteriaceae*, *S. aureus,* sulfite-reducing clostridia and molds and yeast counts were evaluated. Furthermore, pH, water activity and humidity were determined. No growth of *L mocytogenes* was observed during the first 6 days, when the temperature of processing was 4 °C. The low temperature in the initial stages was a relevant hurdle to control *L. monocytoegenes* growth. A significant decrease (*p* ≤ 0.05) in *L. monocytogenes* counts was observed on day 13 compared to the initial counts. During drying (days 6 to 21) a reduction in this pathogen of 1.28 log CFU/g was observed. The low water activity below 0.92 on day 13 and 0.86 on day 21 seems to be critical for the reduction of *L. monocytogenes*.

## 1. Introduction

Chorizo is one of the most popular Spanish dry-cured sausages. Above 30 different kinds of chorizo have been described based on varying methods of production (temperature during fermentation and drying stages, addition or not of starter cultures) and combinations of ingredients [1,2,3]. Riojano Chorizo is a traditional dry cured sausage made in La Rioja, Spain, that obtained the Protected Geographical Indication (PGI) status in 2010 (EC 249/2010) [4]. This kind of chorizo is made from pork fat and lean pork added with sodium chloride, garlic and paprika. Nitrites or sugars are not added to this kind of sausage. The manufacturing process includes low temperatures at the initial stages [3]. Riojano chorizo is often manufactured with traditional technologies without adding starter cultures.

The microbiological quality of Riojano chorizo depends on the combined effect of low water activity, pH and the presence of sodium chloride and other ingredients such as garlic and paprika, which could inhibit undesired bacteria [1]. In addition, the temperatures during fermentation and drying play a role on the microbial populations during the different stages of the process [3]. As sugars and nitrites are not added to Riojano chorizo and the first stages of manufacturing are carried out at low temperatures, its characteristics differ from other types of chorizo. In general, this sausage is recognized as safe, but if pathogens are present in the raw ingredients or contamination occurs during production, and they are able to survive in the end product, then this product could be a risk for consumers [5,6,7]. Few works have studied the microbiological safety of Riojano chorizo [2,8].

*Listeria monocytogenes* and *Salmonella* can contaminate pork meat and may be present in dry-cured meat products [9,10]. Other pathogenic microorganisms such as *Clostridium botulinum*, *Clostridium perfringens*, *Staphylococcus aureus* and *Escherichia coli* have also been found in dry-cured sausages [3,9,10]. During the ripening and drying process, a reduction in pathogen levels has been reported depending on the composition and processing conditions [10].

*L. monocytogenes* causes a severe foodborne disease with a high hospitalization rate (92.1% in the European in 2019) and a high fatality rate (17.6% in the European Union in 2019) [11]. This pathogen can be present in fresh pork meat, ingredients and meat processing environment, and it is able to survive in adverse conditions [7,12] A high prevalence of *L. monocytogenes* has been found in raw meat (45–50%) [13] and food contact surfaces (22.72%) [7]. The contamination of raw meat and processing environment address to the contamination of meat products [12]. In dry-cured sausages prevalence levels of *L. monocytogenes* between 15.8% and 44% have been reported [7,14,15,16]. In consequence there is a great concern in the meat industry on *Listeria monocytogenes* [17]. This bacterium can grow in a wide range of water activity (0.90–0.99), pH (4.4–9.6) and temperature (0–45 °C) conditions [17,18]. Riojano chorizo is a ready-to-eat product, since it does not require any thermal treatment before its consumption. Ready-to-eat meat products, including dry-cured sausages such as chorizo, are often contaminated with *L. monocytogenes* [12,19,20,21,22]. Listeriosis outbreaks have been attributed to the consumption of ready-to-eat meat products [23]. The hazard of the presence of this bacterium tends to decrease during fermentation and drying [24,25], but this pathogen can survive and can be present in the final product [12]. *L. monocytogenes* counts in food should be low in order to ensure the safety of food products. The European Union establishes that the concentration of *L. monocytogenes* in RTE foods that do not support the growth of this pathogen (RTE products with a a_w_ ≤ 0.92 or pH ≤ 4.4 or a_w_ ≤ 0.94 and pH ≤ 5.0) must not exceed 2 log CFU/g [26].

The main natural microflora that can play an important role in dry-cured sausages are lactic acid bacteria, *Micrococcaceae* and in some products also yeasts [27,28]. This microflora are present in raw pork meat and can play a role in controlling the growth of pathogenic bacteria in dry-cured sausages [29,30]. Mesophiles and *Enterobacteriaceae* can be used as indicators of safety in foods [1,2].

The aim of the present work was the evaluate the behaviour of *L monocytogenes* in inoculated Riojano chorizo during manufacturing and drying, as well as the evolution of natural microflora (mesophiles, lactic acid bacteria, *Micrococcaceaee*, yeasts, moulds and *Enterobacteriaceae*).

## 2. Materials and Methods

### 2.1. Bacterial Strains and Inoculum Preparation

Three strains of *Listeria monocytogenes* were used: CECT 934 (serotype 4a), CECT 932 (serotype 1/2a) and CECT 4032 (serotype 4b). The strains were selected considering the serotypes most often involved in listeriosis outbreaks and those most often found in meat processing plants [12,31,32]. A cocktail of the three strains was prepared as follows: each strain was grown in Brain Heart infusion broth (Oxoid, Hampshire, UK) at 30 °C for 18 h as described by Gonzalez-Fandos et al. [3]. Then, the inoculation cocktail was made by mixing equal levels of the individual cultures in 0.1% peptone water (Merck, Darmstadt, Germany) in a sterile container. Appropriate dilutions in the same diluent were prepared in order to obtain a level of inoculum of about 4 log CFU/g in the sausage mixture.

### 2.2. Chorizo Formulation and Processing

Chorizo was made by the traditional production method in the pilot plant of University of La Rioja. A survey in 10 traditional industries included in the PGI was carried out to stablish the processing parameters and composition. Ground pork lean (70%) and pork fat (30%) were supplied by a meat processing industry (La Rioja. Spain). The following ingredients were added per kilogram of meat: 3 g of paprika, 5 g of garlic and 20 g of sodium chloride. After homogenization in a vacuum mixer, the sausage mixture was inoculated with the *L. monocytogenes* strains and mixed for 1 min. A control batch without pathogen inoculation was prepared.

The inoculated sausage mixture was stuffed into 40 mm diameter natural casings in pieces weighing 400 g. After stuffing the sausages were transferred to a drying chamber at 4 °C and 90% relative humidity (RH) for 6 days, then at 13 °C and decreasing RH (90–80%) until day 13 of manufacturing. After, the conditions of the chamber were 15 °C and 80–75% relative humidity. These conditions were maintained until day 21, the RH was reduced gradually a 5% until reaching 75%. Usually, the drying process of Riojano chorizo lasts 21 days. In order to evaluate the effect of a lower water activity, the product was dried an additional week at 15 °C and 70% RH. Samples were taken in the sausage mixture before inoculation and after inoculation, after stuffing and on days 6, 13, 21 and 28 of drying.

Two experiments were carried out. On the sampling days, three samples of sausage mixture or chorizos were taken to perform microbiological analysis and pH, moisture and water activity determinations.

### 2.3. Experimental Design

Two batches of Riojano chorizo were prepared. The first batch was inoculated with *L. monocytogenes*. The second batch was not inoculated with the pathogen. This second batch was used to ensure that *L. monocytogenes* inoculation did not influence the evolution of other microbial groups during processing. For each batch 18 sausages were produced. Samples were taken in the sausage mixture before inoculation and after inoculation, after stuffing and on days 6, 13, 21 and 28 of drying. On the sampling day three samples were taken. A total of 21 samples were taken of inoculated batches consisted of 6 sausage mixtures and 15 sausages. In non-inoculated batches 18 samples were taken: 3 of sausage mixture and 15 sausages. The experiment was repeated twice using two different batches of pork meat.

### 2.4. Physicochemical Analyses

The moisture was measured by drying two homogeneous samples (5 g) at 100 °C to a constant weight. The water activity was determined using an Aqualab TM, 2000 water activity equipment (Aqualab, Madrid, Spain). The pH was measured using a Crison pHmeter with a penetration electrode (Crison Instruments, Barcelona, Spain).

### 2.5. Microbiological Analyses

In this case, 25 g of sausage mixture or chorizo were aseptically weighed and placed in a sterile bag containing 225 mL of sterile peptone water (0.1% *w*/*v*). Then the sample was homogenized in a Stomacher machine (IUL, Barcelona, Spain) for 2 min. Serial decimal dilutions were prepared using the same diluent. Mesophiles were counted on Plate Count Agar (Oxoid) incubated at 30 °C for 3 days [33]. The determination of lactic acid bacteria was conducted in de Man, Rogosa and Sharpe (MRS) agar (Oxoid) after incubation at 30 °C for 3 days [34]. The enumeration of *Micrococcaceae* was conducted in Manitol Salt agar (Oxoid) with incubation at 37 °C for 2 days [2]. Yeasts and moulds counts were determined in oxytetracycline glucose-yeast extract agar (Oxoid) after incubation at 25 °C for 3–5 days [2]. Sulfite-reducing clostridia were evaluated using sulfite polymyxin sulphadiazine agar (Oxoid) incubated at 37 °C for 24 h under anaerobic conditions [35]. *Staphylococus aureus* was enumerated on Baird Park agar containing egg yolk tellurite (Oxoid) incubated at 37 °C for 48 h [36]. Suspected colonies were tested for coagulase production [36]. *Enterobacteriaceae* were enumerated on plates of Violet Red Bile Glucose agar (Oxoid), the plates were overlaid before the incubation at 37 °C for 1 day [37]. Results were expressed as log_10_ CFU/g.

For the determination of *L. monocytogenes* 25 g of chorizo were weighed aseptically and homogenised with 225 mL of half Fraser broth (Oxoid). Serial decimal dilutions were prepared in half Fraser broth, 0.1 mL samples of appropriate dilutions were spread onto chromogenic Listeria agar ALOA plates (BioMerieux, Marcy l’Etoile, France). Plates were incubated at 37 °C for 2 days. Presence/absence of the pathogen in 25 g was performed according to the following procedure: After a pre-enrichment in 225 mL of half Fraser broth (Oxoid) at 30 °C for 24 h, 0.1 mL samples were inoculated into 10-mL tubes of Fraser broth (Oxoid) and incubated for 48 h at 37 °C. This secondary enrichment culture was streaked onto chromogenic Listeria agar (BioMerieux) and incubated at 37 °C for 48 h [38,39]. Suspected colonies were identified according to the method described by Gonzalez-Fandos and Herrera [40]. The enumeration and presence/absence of *L. monocytogenes* was also evaluated in ground pork meat and fat, and in paprika and garlic.

Growth parameters (maximum growth rate and lag phase) were obtained using the program DMFit [41].

### 2.6. Statistical Analysis

Analysis of variance was performed using the statistical package Statgraphics (Centurion XVIII, Statgraphics.Net, Madrid, Spain). Tukey’s test for comparison of means was performed using the same program. Plate count data were converted to logarithms prior to their statistical treatment. The experiments were replicated twice on different occasions with different ground meat samples. The analyses were run in triplicate for each replicate. Significance level was defined at *p* ≤ 0.05.

## 3. Results

Table 1 shows the pH, water activity and moisture content during the manufacturing process of inoculated chorizo. The pH values of the sausage mixture before inoculation were 5.90 ± 0.01, remaining constant after the stuffing on day 0. The pH values decreased slightly during the drying process until day 13, then a slight increase was observed. The water activity and moisture remained constant in sausage mixture and after stuffing (day 0). The moisture decreased from an initial value after stuffing of 52.00% ± 0.22 to 33.96% ± 0.52 on day 21. The initial water activity after stuffing was 0.965 ± 0.003, reaching a value of 0.860 ± 0.005 on day 21. During the first 6 days the water activity decreased from 0.965 ± 0.003 to 0.942 ± 0.004. During the following period until day 13 the water activity decreased to 0.912 ± 0.006. On day 28 a significant decrease (*p* ≤ 0.05) in water activity was observed compared to day 21, with values of 0.793 ± 0.004 on day 28. No significant differences (*p* > 0.05) in water activity, pH or moisture content were observed between the chorizos inoculated with *L. monocytogenes* and those not inoculated (data not shown).

**Figure 1:**
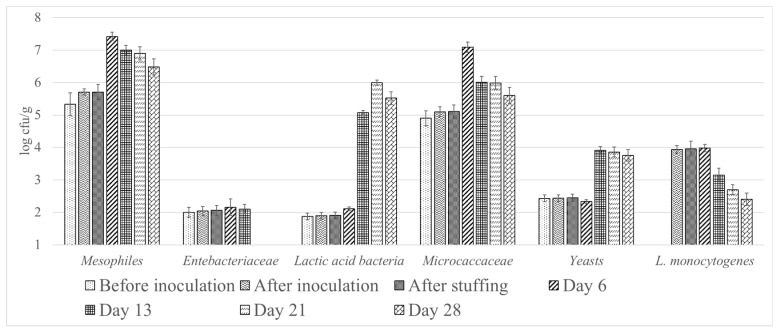
Evolution of the microbial groups in chorizo inoculated with *L monoocytogenes* during manufacturing.

Figure 1 shows the evolution of different microbial groups throughout the process. No significant differences (*p* > 0.05) were observed in mesophiles, lactic acid bacteria, *Micrococcaceae*, yeast and *Enterobacteriaceae* counts among samples analysed before inoculation, after inoculation and after stuffing. After inoculation (day 0) the mesophiles counts were 5.33 ± 0.35 log CFU/g, the highest mesophiles counts were observed on day 6 (7.42 ± 0.13 log CFU/g), after a progressive slight decrease was observed. The initial lactic acid bacteria counts were 1.90 ± 0.10 log CFU/g, a significant increase was observed on day 13 reaching telvels of 5.07 ± 0.07 log CFU/g. The highest lactic acid bacteria counts were observed on day 21 (6.00 ± 0.08 log CFU/g). The initial *Micrococcaceae* counts were 5.10 ± 0.15 log CFU/g. The highest *Microccocaceae* counts were observed on day 6 (7.09 ± 0.16 log CFU/g). The levels of *Microccocaceae* in the final product were 5.99 ± 0.20 log CFU/g. The populations of lactic acid bacteria and *Micrococacceae* decreased slightly if the sausages were dried an additional week (day 28). The initial yeast count were 2.44 ± 0.01 log CFU/g, a significant increase was observed on day 13, reaching levels of 3.91 ± 0.15 log CFU/g, these populations remained almost contant until the end of the drying period. *Enterobacteriaceae* decrease throughout the process. After inoculation the levels of *Enterobacteriaceae* were 2.04 ± 0.14 log CFU/g. The levels in the final product were less than 1 log CFU/g. Sulfite-reducing clostridia, *S. aureus* and moulds were not detected in any sample analysed. No significant differences (*p* > 0.05) were observed in mesophiles, lactic acid bacteria, *Micrococcaceae*, yeast and *Enterobacteriaceae* between chorizos inoculated and not inoculated on the same sampling day (data not shown).

*L. monocytogenes* was not detected in the sausage mixture before inoculation. No presence of *L. monocytogenes* was detected in ground meat, pork fat or ingredients used for the manufacture of chorizo. After inoculation, the *L. monocytogenes* counts were 3.94 ± 0.12 log CFU/g. In the fresh stuffed chorizo the counts were 3.96 ± 0.23 log CFU/g. The water activity of the fresh stuffed chorizo was 0.965 and the humidity 52% and the initial pH was 5.93.

On day 6 of elaboration, the *L. monocytogenes* populations were similar to the initial ones, no growth neither inactivation were observed. On day 13 a significant decrease of 0.79 logarithmic units compared to the initial levels was observed. At the end of the drying process (21 days) the decrease in the *L. monocytogenes* population was 1.24 log units. When drying was carried out for one additional week (day 28), a reduction of 1.54 logarithmic units was obtained compared to the initial level.

The growth parameters (maximum growth rate and lag phase) of *Listeria monocytogenes*, lactic acid bacteria and *Micrococcaceae* estimated by the program DMFit are shown in Table 2. An extended lag phase for lactic acid bacteria (6.539 days) and *L. monocytogenes* (5.212 days) was observed, while a shorter lag phase for *Micrococcaceae* was found (0.899 days). The maximum growth rate for *L. monocytogenes* was negative (−0.093 log cfu/g/day) indicating an inactivation of the pathogen. The maximum growth rate for lactic acid bacteria was higher than that observed for *Micrococcaceae*. The R^2^ and SE values obtained were above 0.90 and below 0.350, respectively.

## 4. Discussion

Similar pH values of meat batter were reported by Fonseca et al. [42] (5.95) in Galician chorizo and by Christieans et al. [5] (5.85 to 5.94) in saucisson. In addition, Chevallier et al. [5] obtained pH values between 5.8 and 6 in the meat batter collected in a processing plant of traditional dried sausages. As in the present work, Christieans et al. [5] also obeserved that pH remained constant after stuffing. However, these authors observed a pH decreased during the first 6 days, reaching a value of 5.05 ± 0.05 on day 6. In the present work a slight decrease was observed in the first days, reaching a value of 5.88 ± 0.03 on day 6. These differences could be explained since Christieans et al. [5] added sugars and starters in the process, while no sugars or starters were added in the present work. As in the current study Fonseca et al. [42] and Christieans et al. [5] observed a slight increase in the pH during the end of the drying period. The pH of Riojano chorizo at the end of the drying process (5.78 ± 0.02 on day 21) is higher than that found in other types of chorizo in the literature (below 5.3) [20,43,44,45,46]. The little decrease in pH compared to other types of chorizo is due to the fact that no sugars are added to Riojano chorizo. In addition, high pH values at the end of drying have been reported in chorizo by Fonseca et al. [38] and in salami by Tirloni et al. [47] (5.42–5.55) and Bellegia et al. [48] (5.49–5.69). As in the present work also Bellegia et al. [48] reported that for the production of Ciascolo salami drying was carried at low temperatures: at 4 °C for 6 days at 85%RH, and after at 10 C for 20 days and 82% RH. These authors reported pH values of 5.86–5.87 on day 0, 5.66–5.75 on day 5 and 5.49–5.69 on day 20.

Similar moisture content in chorizos on day 0 was reported by Beriain et al. [49] (50.94%. 51.57% in the present work) and 26.57% on day 31 (25% on day 28 in the present work). Similar a_w_ values in initial meat batter were reported by Christieans et al. [5] (0.97 and 0.96 in the present work). However, at the end of drying of saucisson (21 days) the a_w_ found by Christieans et al. [5] was higher (0.89–0.90) compared to the present study (0.860 ± 0.005), since the conditions of temperature and relative humidity were different in both studies. In addition, higher a_w_ values (0.954) were observed by Bellegia et al. [48] in salami dried at low temperatures, but higher relative humidity. Fonseca et al. [42] reported similar a_w_ values at the end of drying 0.856 on day 21 and 0.83 on day 30. Riojano chorizo is a low-acid dry sausage (pH above 5.6) [26]. In contrast, the pH of high acid sausages is lower than 5.3 and can be 4.5. Since the pH of the end Riojano chorizo is relatively high, the a_w_ is a critical factor to control the microbial growth.

Higher initial mesophiles counts on day 0 (6.3–6.8 log cfu/g) were reported by Benito et al. [50] in Iberian chorizo without addition of starters (5.45 in the present work). These authors also observed an increase in mesophiles counts during ripening (1.1–2 log units after 60 days) and a slight decrease at the final stage of ripening with populations of 6.1–7.8 log cfu/g. Similar initial mesophiles counts (5.24 log CFU/g) were found in Galician chorizo [42]. Lower mesophiles counts in final chorizo were reported by Cava et al. [32] (about 5.5 log cfu/g).

Higher initial *Enterobacteriaceae* counts on day 0 (4.1–5.2 log cfu/g) were reported by Benito et al. [50] in Iberian chorizo without addition of starters (2.06 in the present work). *Enterobacteriaceae* low counts are related to good hygiene conditions [51]. As in the present study, these authors observed a reduction of *Enterobacteriaceae* counts during ripening, being the levels in the final product below de detection limit (<1 log CFU/g). In addition, other authors have reported a decrease in *Enterobacteriaceae* population during the processing of dried-cured sausages [48,51]. Benito et al. [50] attributed the reduction of *Enterobacteriaceae* to the decrease of the pH values, since only a slight decrease of pH was observed in the present work the reduction of *Enterobacteriaceae* could be related to other factors such as the low temperatures, ingredients (garlic, paprika) the decrease of a_w_ and the competitive microflora (*Micrococcaceaea* and lactic acid bacteria). As in the present work other authors have not detected moulds in other dry-cured sausages with short ripening periods [48].

The dominant flora in dry-cured sausages is represented by lactic acid bacteria and *Micrococcaceae*, which may have an important technological role in these meat products [51]. Lactic acid bacteria are relevant since they play a role in the fermentation process and preservation of sausages, their contribution relies mainly in the production of organic acids and volatile compounds through the fermentation of sugars and the consequent decrease of pH [28] *Microcaccaceae* produce proteases and lipases and have the ability to reduce nitrates, being involved in the aroma and colour development [52].

Higher initial lactic acid bacteria counts (4.1–4.86 log CFU/g) were reported by Benito et al. [50] in Iberian chorizo, although no starter was added (1.9 ± 0.1 log CFU/g in the present work). Fonseca et al. [42] reported initial lactic acid bacteria counts of 3.28 log CFU/g. In addition, higher lactic acid bacteria counts between 2.95 and 3.30 log CFU/g have been reported by other authors in other dry-cured sausages [49]. Differences in initial lactic acid bacteria counts can be explained since some processing methods keep the sausage mixture during 24–48 h at temperatures of 10 °C or below, and the samples were taken after this period [20]. However, Chevallier et al. [43] reported lactic acid bacteria counts of 1.91 log CFU/g in samples of meat batter collected in winter in a processing plant of traditional dry-cured sausages, whereas the counts were 3.94 log CFU/g in the batter samples collected in spring. According to Chevallier et al. [43] differences in temperatures of the processing plant in spring and winter influenced the microbial counts of raw meat. Benito et al. [50] observed an increase of lactic acid bacteria populations during ripening reaching levels about 8 log CFU/g after 60 days, and a slight decrease at the end of ripening (day 120) with populations of 7.2–7.6 log CFU/g. The conditions of fermentation in the mentioned work were temperatures between 18–20 °C during the first 2–3 days and 85% RH, after the temperature was 10–12 °C and the ripening process was longer (120 days) than in the present study (21 days) [50]. We observed a significant increase in lactic acid bacteria after day 6, when temperature was changed from 4 °C to 15 °C, with an increase of 2.96 log units between day 6 and 13. This fact could be explained since some strains of lactic acid bacteria are not able to grow at 4 °C, but they can grow at 15 °C [50]. Other authors have also reported that the counts of lactic acid bacteria did not increase during the first 5 days in salami at temperature of processing of 4 °C, while growth was observed in the following stage when temperature was increased to 10 °C [47]. These results are in agreement with those obtained in the present work, since we also observed growth of lactic acid bacteria when the temperature of processing was increased. On the other hand, lactic acid bacteria are able to grow at low water activity values, they can grow at values of a_w_ above 0.79, but a high percentage are not able to grow at a_w_ of 0.75 [50]. A slight decrease in lactic acid bacteria counts was observed on day 28 (6 log CFU/g). This decrease can be related to the decrease in a_w_ from 0.86 to 0.79. In the present work no sugars neither nitrites were added, while Benito el al. [50] added sugars and nitrites. Since sugars were not added the pH values were higher in the present work. The low growth rate of lactic acid bacteria could be the result of the low temperatures used as well as the no addition of sugars [42]. Lower final counts of lactic acid bacteria were observed in the present work compared to those reported by Gonzalez-Fandos et al. [2] in chorizo. These differences could be explained by the lower drying temperatures.

In the current study the initial *Micrococcaceae* counts (5.10 log cfu/g) were higher than those reported by other authors [50,53]. Similar initial counts were pointed out by Cardinali et al. [54] in salami (4.78–5.05 log cfu/g) and Fonseca et al. [42] in chorizo (4.99 log CFU/g). In contrast, Benito et al. [50] found lower initial *Micrococaceae* counts in Iberian chorizo elaborated without starter (2.1–2.6 log CFU/g). These authors observed an increase on day 60 reaching populations of 4.3–4.8 log CFU/g, at the end of ripening (120 days) this microbial group remained stable (4.2–4.6 log CFU/g) or decreased (2.9–3.8 log CFU/g), depending on the industry where the chorizos were produced. In the present work on day 13 a decrease in *Micrococcaceae* counts was observed, remaining stable until the end of the process, on day 13 the a_w_ was below 0.92 [50]. In the present work the dominant microflora on days 6 and 13 was the *Micrococcaceae* instead of lactic acid bacteria. On days 21 and 28 similar populations of *Micrococcaceae* and lactic acid bacteria were observed, around 6 log CFU/g. *Micrococcaceae* counts were maintained until the end of the process. Christieans et al. [5] reported a fast increase of lactic acid bacteria on the first 6 days in French dry-cured sausages, these levels remained stable during day 6 and 21. These authors added starters to the meat batter with an inoculation level of lactic acid bacteria of 6 log CFU/g, reaching populations of 8.5–9 log CFU/g [5]. However, *Micrococcacease* inoculated at a level of 6 log CFU/g, only grew slightly during the first 6 days, reaching a value of 6.7 log CFU/g on day 6 and after a slight decrease until day 21 [5]. In contrast in the present work, a higher increase of *Micrococcaceae* were observed on the first 6 days, reaching a value of 7.09 log CFU/g. As Christieans et al. [5] a slight decrease in *Micrococcaceae* counts was observed between day 6 and 21. We found a slight increase in lactic acid bacteria counts on the first 6 days. However, a higher increase was observed between day 6 and 21. Other authors have reported that lactic acid bacteria is the dominant microflora in chorizo [50,55]. On the other hand, some strains of *Micrococcaceae* are sensitive to low pH, but able to adapt to the low temperatures of ripening, being the dominant microflora in some dry-cured sausages with relatively high pH [56]. Thus, Samelis et al. [53] reported an increase in *Micrococcaceae* counts of 2 log in salami batches with low initial counts of lactic acid bacteria, while the increase was about 1 log in the batches with higher lactic acid bacteria counts. *Microccaceae* are poor competitors in the presence of actively growing lactic acid bacteria [57]. Thus, Gonzales-Barron et al. [57] observed that *Micrococcaceae* were more competitive in dry-fermented sausages with a delay in acidification, under these conditions these microorganisms increased 2 log CFU/g from 3.86 in sausage mixture to 5.93 log CFU/g in ripened sausages, whereas in the sausages with a good acidification profile only an increase of 1 log CFU/g was found. Cardinali et al. [54] reported that *Micrococcaceae* are present at all the stages of salami ripening, being the dominant group at the end of ripening in those sausages where nitrites were not added. Hospital et al. [58] observed that in the absence of nitrites *Micrococcaceae* counts were 2 log CFU/g higher than in the presence of nitrites. However, other authors have reported that Micrococcaceae are not affected by nitrites [5].

Yeast levels are similar to those found by other authors in chorizo (between 3 and 5 log CFU/g). The initial yeast counts observed by Bellegia et al. [48] in salami (3.81–3.97) were higher than in the present work (2.45 CFU/g). As in the present work Bellegia et al. [48] did not observe any increase in yeast counts during the first 5 days when temperature was 4 °C. When temperature increased (10 °C), these authors observed a progressive increase in yeast counts, with an increase of 1.55 log CFU/g on day 10 in one of the batches produced. In the present work on day 13 an increase of 1.58 log CFU/g was observed. Levels of yeast in final product about 2–4 log cfu/g has been found in other traditional dry sausages [19,43,59]. Cava et al. [32] found similar yeast counts in chorizo at the end of the process, about 3.5 log cfu/g. (3.76 log CFU/g in the present work). The presence of yeasts in different kinds of chorizo suggests their participation in the process [27].

In the present work no growth of *L. monocytogenes* was observed in any stage of processing. However, other authors have reported that *Listeria monocytogenes* can grow in dry-cured sausages during processing [5,20,60,61]. In the current study no growth of *L. monocytogenes* was observed between day 0 and day 6, when a_w_ was high (0.965 on day 0 and 0.941 on day 6) and also pH (5.93 on day 0 and 5.88 on day 6), but processing temperature was low (4 °C). In contrast, Christeans et al. [5] observed an increase of *L. monocytogenes* growth from 2 log CFU/g (day 0) to 3.5 log (day 6) in saucisson with similar initial a_w_ and pH (0.97 and 5.94, respectively), but higher processing temperatures (24 °C). However, Christeans et al. [5] observed that in presence of nitrite the initial *L. monocytogenes* counts remained without changes during the first 6 days. A decrease of *L. monocytogenes* counts was reported by Christieans [5] during the drying period (38 days) at 13–14 °C and RH 80–82%, with pH and a_w_ values of 5.3–5.4 and 0.89–0.90, respectively. As in the present work, a decrease of 0.5 log CFU/g on day 34 was observed in saucisson elaborated with nitrate, whereas a reduction of 1 log CFU/g was observed in those in which nitrite was added. Degenhardt et al. [60] observed a small increase in the counts of *L. monocytogenes* in the first days of processing in natural contaminated sausages at temperatures of 22–24 °C and RH 94–98%. In addition, Enicinas et al. [20] reported that *Listeria monocytogenes* can be present in chorizo formulations, being able to grow in the first stages and survive the drying process. Initial levels of *Listeria* spp. of 1.17 log CFU/g in natural contaminated chorizo elaborated without starter and ripened under natural conditions were reported by Encinas et al. [20]. These authors observed an increase of the pathogen of 1.47 log CFU/g on day 11, and then a decrease reaching populations similar to the initial ones (1.44 log CFU/g and 1.54 log CFU/g on days 25 and 32, respectively). The pH of chorizos was 5.01 on day 11 and 4.80 on day 25 and the lactic acid bacteria counts were 8.40 log CFU/g on day 11 and 8.95 log CFU/g on day 25. However, in chorizos elaborated without starter and ripened at low temperatures (10–14 °C) and RH (70–80%) the initial *Listeria* spp counts (3.5 log CFU/g), remained almost constant until day 18 and after decreased 0.5 log CFU/g on the final product (day 32). Garriga et al. [61] reported a significant increase in *L monocytogenes* counts during the first 7 days of ripening in fuel and chorizo elaborated without starter and dried at 12 °C and RH 95% (at the beginning of the process and gradually reduced to 80% until day 10). However, these authors did not observed growth of the pathogen when starter cultures were added. According to Nightingale et al. [62] the Italian-style salami manufacturing process with fermentation at 30 °C for 24–72 h, and after drying at 10–13 °C and 75–85% RH was relatively ineffective at reducing *L. monocytogenes* populations, with reductions below 1.0 log CFU/g. As in the present work, Novelli et al. [63] suggested that the cold conditions during the drying play a protective role against *L. monocytogenes*. In addition, Branciari et al. [64] reported a negative maximum growth rate for *L- monocytogenes* in salami, although lower than in the present work (−0.0456 log CFU/g/day). In the present study the overall fitting of data using DMfit was generally good, with R^2^ and SE values above 0.90 and below 0.350, respectively. The R^2^ and SE values obtained by Branciari et al. [64] for *L- monocytogenes* growth in salami were 0.743 and 0.25, respectively, while in the present work were 0.910 and 0.216. These results indicate that the growth of *L. monocytogenes* was suppressed in Riojano chorizo.

Lebert et al. [19] studied the microbiology of natural dry fermented sausages from nine processing plants. These authors pointed out that a high variability in the ingredients and fermentation and ripening conditions. The temperatures during fermentation ranged between 11 and 20 °C and the relative humidity between 76% and 99% during a period of 2–8 day. The temperature during ripening ranged between 8 and 14 °C and relative humidity between 70% and 90% during 8 to 14 days. In three of the plants the ripening was carried out under natural conditions. Three of the plants did not add sugars. The pH of the final meat products in which sugars were not added ranged between 5.76 and 6.39. In general, lower pH values (5.21–5.79) were observed in those sausages in which sugars were added, except in one case with a pH value of 6.22. Sugars enhance the growth of lactic acid bacteria, which produce lactic acid and decrease the pH. These pH (5.21–6.39) are characteristic of low or slight acid dry fermented sausages manufactured in Mediterranean countries. Lebert et al. [19] detected *Listeria monocytogenes* in three of the nine processing plants studied, the pathogen was detected in the final product at levels below 2 log CFU/g in two of the plants, whereas in one processing plant with a pH in the final product of 6.39 the levels detected were 2.8 log CFU/g. The results obtained in the present study are in accordance with those reported by Garcia-Diez et al. [25], who observed a decrease of *L monocytogenes* inoculated in chorizo without starter and sugars during the drying period, from an initial level of 2.33 ± 0.29 on day 1 to 0.95 ± 0.66 on day 15.

Mataragas et al. [65,66] pointed out the inactivation rate of *L monocytogenes* in inoculated salami as the result of the pH, aw, and the fermentation temperature. These authors only observed small reductions of this pathogen lower than 1 log CFU/g (0.38–0.39 log CFU/g), mainly attributed to the slow decrease in the aw values, with a final a_w_ above 0.93 and fermentation temperature above 20 °C). The pH although decreased relatively fast, its contribution to this pathogen reduction was limited. According to Mataragas et al. [65,66] the environmental conditions of the first 48 h are critical for the growth and survival rate of this pathogen.

According to Gounadaki et al. [67] at high fermentation temperatures (above 20 °C), the critical factor for *L. monocytogenes* reduction is the pH decrease, whereas at lower temperatures the critical factor is the a_w_ reduction. When this reduction is slow, *L. monocytogenes* may survive longer. Our results showed similar pH values in the final product to those found in other Mediterranean dry cured sausages (5.4–5.8). The pH values were within the range of growth of *L. monocytogenes*, being the drying process critical to minimize the potential for *L. monocytogenes* growth [10]. Ruggeri et al. [68] reported that *L. monocytogenes* is able to grow in fermented sausages dried at 20–22 °C the first days and after at 15 °C only when a_w_ was above 0.92. As in the present study, other authors have also pointed out the importance of a*_w_* in dry-cured sausages to control *L. monocytogenes* [57,60,62,63,65,67,68].

Another important factor that could affect the behaviour of *L. monocytogenes* in dry-cured sausages is the presence of indigenous microflora [30,59,69]. Several works have shown that lactic acid bacteria may inhibit the growth of *L. monocytgenes* by means of antimicrobial metabolite production, competition for nutrients and microbial antagonism [30]. According to some authors the lactic acid bacteria present in dry-fermented sausages exerts an inhibitory effect on *L. monocytogenes* growth, mainly when the levels of this microflora is present at levels above 4.5 log CFU/g [29,30]. We observed these levels of lactic acid bacteria when reductions of the pathogen were found. Moreover, an extended lag phase for lactic acid bacteria (6.539 days) and *L. monocytogenes* (5.212 days) were observed. After, the maximum growth rate for lactic acid bacteria was 0–500 log CFU/g/day, while a negative value for *L. monocytogenes* was observed (−0.093 log CFU/g/day), indicating an inactivation of the pathogen, when growth of lactic acid bacteria was observed. On the other hand, some studies have shown that *Micrococcaceae* can inhibit the growth of pathogens in dry fermented sausages, including *L. monocytogenes* [70,71,72]. Villani et al. [72] reported that *Staphylococcus xylosus* reduced the counts of *L. monocytogenes* by 2 log units in salami after 21 days. We observed that the *Micococcaceae* constituted the major microorganism of the chorizo on day 6, since the number of mesophiles and *Micrococcaceae* were very similar on day 6 of drying. Some authors have reported that yeasts may have an antagonist effect against *L. monocytogenes* [73].

It should be noted that paprika and garlic used in the preparation of Riojano chorizo contain phenolic compounds and other bioactive substances with potential antioxidant and antimicrobial properties [74].

As mentioned, the differences found in the effectiveness on the manufacturing process to control *L* *monocytogenes*, can be explained by the different process parameters that change along the ripening period (temperature, relative humidity), ingredients (paprika, garlic), preservatives (nitrites) and the competitive flora present (lactic acid bacteria, *Micrococcaceae*).

The results *obtained* in chorizo show that during drying *L. monocytogenes* counts decreased, but this pathogen can survive, being found in the end product. Some researchers have pointed out that depending on the initial contamination levels *Listeria monocytogenes* could not be completely eliminated during the drying process [62,75]. Johnson et al. [75] observed that *Listeria monocytogenes* did not grow during fermentation and drying of salami, but if it is initially present above 3 log CFU/g survival may occur, and the pathogen could be present in the final product. Normally, *L. monocytogenes* contamination in raw meat is below 2 log CFU/g [15,21]. In our study the *L. monocytogenes* inoculum was higher than these levels found in the naturally contaminated meat (4 log CFU/g), and the pathogen was detected in the final product. As suggested by other authors, the final levels of *L. monocytogenes* in dry-cured sausages and the compliance of the EC Regulation of microbiological criteria (<2 log CFU/g at the time of consumption in those products that no support the growth of *L. monocytogenes*) [26] mainly depends on the initial contamination level [76] and on the growth potential in the first processing stages when pH and aw values are high [45]. Our results indicated that in the initial stages the low temperatures (4 °C) did not promote the growth of *L. monocytogenes*. Therefore, it is necessary to control the initial levels of the pathogen in meat and other raw ingredients as well as in the equipment used.

## 5. Conclusions

The results of the current work indicate that *L. monocytogenes* is unable to grow during the manufacturing process of Riojano chorizo. Even more, reductions on this pathogen counts were observed when a_w_ was low (days 13 to 28). The results obtained pointed out that the initial stages are critical to avoid the growth of *L. monocytogenes* in Riojano chorizo. The low temperature (4 °C) in the initial stages is a relevant hurdle to control this pathogen when pH and a_w_ are high. During drying the low water activity is a critical factor to control *L. monocytogenes*. The Riojano chorizo manufacturing process is not able to inactivate completely *L. monocytogenes* if the microorganism is initially present at levels of 4 log CFU/g. Therefore, it is necessary to limit the initial contamination in meat and other raw ingredients, as well as in the equipment used. Special care must be taken to ensure that cleaning and disinfecting procedures are applied correctly. Hazard Analysis Critical Control Point techniques should be applied in order to identify and control sources of *L. monocytogenes* contamination and dissemination.

## Figures and Tables

**Table 1:** Physicochemical parameters of inoculated Riojano chorizo during processing.

Stage	pH	Water Activity	Moisture (%)
Sausage mixture before inoculation (day 0)	5.90 ± 0.01 ^a^	0.960 ± 0.005 ^a^	51.57 ± 0.12 ^a^
Sausage mixture after inoculation (day 0)	5.92 ± 0.01 ^a^	0.969 ± 0.002 ^a^	51.68 ± 0.15 ^a^
After stuffing (day 0)	5.93 ± 0.02 ^a^	0.965 ± 0.003 ^a^	52.00 ± 0.22 ^a^
Day 6 of drying	5.88 ± 0.03 ^a^	0.942 ± 0.004 ^b^	46.60 ± 0.54 ^b^
Day 13 of drying	5.72 ± 0.04 ^b^	0.912 ± 0.005 ^c^	39.94 ± 0.65 ^c^
Day 21 of drying	5.78 ± 0.02 ^b^	0.860 ± 0.005 ^d^	33.96 ± 0.52 ^d^
Day 28 of drying	5.79 ± 0.01 ^b^	0.793 ± 0.004 ^e^	25.00 ± 0.62 ^e^

Results expressed as mean ± standard deviation (*n* = 6). Means with different superscript letters within the same column were significantly different (*p* ≤ 0.05).

**Table 2:** Growth parameters of *Listeria monocytogenes*, lactic acid bacteria and *Micrococcaceae*.

Microoganism	ʎ	µ_max_	R^2^	SE (Fit)
*L. monocytogenes*	5.212	−0.093	0.910	0.216
Lactic acid bacteria	6.539	0.500 ^a^	0.970	0.339
*Micrococcaceae*	0.899	0.339 ^a^	0.981	0.131

ʎ, lag phase (day), µ_max_, maximum growth rate (log CFU/g/day), R^2^, coefficient of determination, SE (fit), Standard error of fit.

## Data Availability

Research data are not share.
